# The partner-an underutilized facilitator to support healthy gestational weight gain

**DOI:** 10.1186/s12884-023-05715-1

**Published:** 2023-06-15

**Authors:** Joshua R. Sparks, Suzanne Phelan, Kimberly L. Drews, Leanne M. Redman

**Affiliations:** 1 Pennington Biomedical Research Center, Louisiana State University, Baton Rouge, LA 70808 USA; 2 California Polytechnic State University, San Luis Obispo, CA 93407 USA

**Keywords:** Barriers, Facilitators, Gestational weight gain (GWG), Health behaviors, Partner, Pregnancy

## Overview

Addressing barriers and facilitators to health behavior changes for support of optimal gestational weight gain (GWG) in pregnancy is a cornerstone for the development and implementation of prenatal interventions. Most research and interventions to date, however, are focused solely on the pregnant women. According to the Social Ecological Model of Behavior Change, support persons and the immediate household environment are crucial pillars of behavior change. In pregnancy, the non-pregnant partner offers an avenue for support and opportunity to influence the household environment in a meaningful way. In this Letter to the Editor, we acknowledge the importance of the paper by Escañuela Sánchez et al. entitled *“Facilitators and barriers influencing weight management behaviours during pregnancy: a meta-synthesis of qualitative research”*. The authors underscored the limited knowledge the field has regarding partner engagement and, moreover, identified that the partner is a critical stakeholder to optimize not only maternal health, but paternal health and long-term household behaviors during this optimal period of human development.

## Body of work

Barriers and facilitators to promote recommended gestational weight gain (GWG) are principal considerations in behavioral interventions targeting improvements in health behaviors of women during pregnancy. To date, most prenatal behavioral interventions have targeted the pregnant woman only. While logical, this approach may lessen the impact of behavior change strategies to foster healthy GWG even when considering appropriate individual-level barriers and facilitators.

**Fig. 1:**
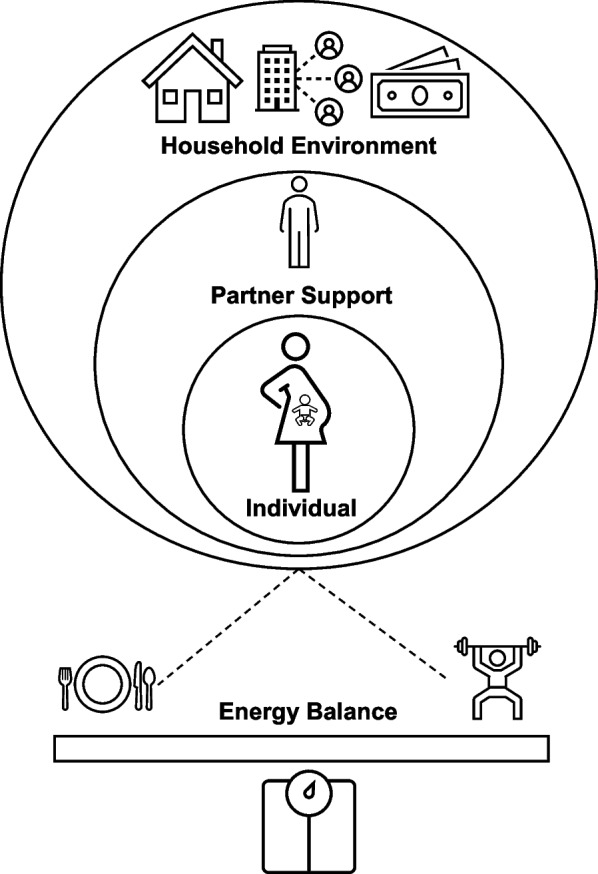
Adapted ecological model of behavior change and maternal GWG. Behavior change depends on individual-level factors and is influenced by partner support and the household environment, which may impact health behaviors and GWG

The Social Ecological Model of Behavior Change [1] posits that individual behaviors have multiple levels of influence, such as intrapersonal (biological, psychological), interpersonal (social, cultural), organizational, community, physical, environmental, and policy. In the context of pregnancy, we hypothesize that promotion of effective behavior changes to support healthy GWG depends on individual-level factors, in addition to support and adoption of health behavior change in partners, and couple-determined changes to the overall household environment (Fig. 1).

We read the study by Escañuela Sánchez and colleagues with great interest [2]. Using a meta-synthesis of qualitative research reporting barriers and facilitators influencing weight management behaviors during pregnancy, the authors concluded that interventions aimed to promote and maintain weight management behaviors during pregnancy should consider all levels of influence in shaping a woman’s behaviors. They suggested input from multiple stakeholders in the pregnancy, including extended family members and the social network. However, the authors also noted that family members can negatively influence a woman’s ability to make behavior change and promote detrimental health behaviors, such as overeating and sedentary behavior.

To our knowledge, and as pointed out by the authors, this is the first meta-synthesis of qualitative data to understand the best means to promote healthful GWG from the viewpoint of the pregnant woman. The body of work summarized up through March 2019 and updated in January 2021 highlights three important observations which underscore the need for future research to understand barriers and facilitators of partners, and that future prenatal interventions should target the pregnant couple collectively.

First, their reported observation that most women had no decision making power over shopping or cooking choices in the family was surprising. While reports often tout the mother as the ‘nutrition gatekeeper’ of the home, this may not be the rule. In many modern households, traditional housekeeping roles are shared among both partners [3] and, in multi-generational households, meals and food considerations may reside in other family members [4]. These factors were not noted in the present meta-synthesis and are a missed opportunity to recognize the influence of the partner and their needed support. A cross-sectional study suggested that increased familial support for a healthy diet predicted greater intake of fruits and vegetables consumed in pregnant women [5].

Second, their statement that partners encouraged pregnant women to overeat and that some women reported their partners as a ‘feeder’ suggests partners have their own view of eating behavior that might change during pregnancy. While there is a social dogma that supports ‘eating for two’ in pregnancy, our work shows that excess GWG is largely the result of excess energy intake [6, 7]. Partners engaged in prenatal interventions have the potential to learn alongside the pregnant woman regarding the value of optimizing nutrition in pregnancy while maintaining minimal increases to overall calorie intake. Further, in couple-based interventions, the duo can work together to set goals and hold each other accountable through healthy meal planning and preparation of shopping lists in the context of the household budgets, needs, and desires.

Third, the authors described that some women reported relying on family and friends for advice on healthful diets and physical activity; yet this advice was sometimes discordant with their own beliefs. While not discussed in the paper, this observation underscores the need to consider the couple as a whole in future pregnancy interventions. Studies suggests that couple concordance in healthy eating habits is most impactful for achieving recommended GWG [8]. Additionally, previous evidence suggests that partners are critical facilitators to physical activity engagement during pregnancy to promote healthy GWG, including couple concordance in physical activity behaviors [9]. Promoting recommended GWG in mothers alone has had little and no effect on partner weight changes to date [10, 11]; but, targeting both the pregnant woman and her partner may have the synergistic effect of optimizing outcomes in both the couple and other family members in the household. Taken together and to advance we field forward; we need to understand the partner’s perceived barriers and facilitators for the pregnant woman to adopt health behavior change and how to actively engage the partner in prenatal interventions to optimize overall health behaviors within the household.

## Conclusion

In conclusion, we commend the authors for synthesizing the available qualitative literature to establish key individual-level barriers and facilitators influencing weight management behaviors during pregnancy. We suggest an additional conclusion that may be drawn is to recognize the essential role of the non-pregnant partner during pregnancy and the exciting future direction of this field to develop couple-based prenatal interventions. The partner is a key stakeholder, closest support person, and may act as a core facilitator to implement appropriate behavior change to promote healthful and recommended GWG. Completed trials from our group suggest that 67–76% of women who enroll in prenatal interventions identify as being married and/or in a domestic partnership, not including those with an active partner in pregnancy [12, 13]. As such, there exists the opportunity to engage partners in pregnancy into prenatal interventions aimed at meeting recommendations for GWG and optimizing health outcomes for women and children.

## Data Availability

Not applicable.

## Abbreviation

GWG: Gestational Weight Gain
